# Renoprotective effects of remote ischemic preconditioning on acute kidney injury induced by repeated tourniquet application in patients undergoing extremity surgery

**DOI:** 10.3389/fmed.2024.1477099

**Published:** 2024-12-20

**Authors:** Ziying Tao, Yang Zhang, Erliang Kong, Haili Wei, Mingyue Li, Shuhui Sun, Liwei Liu, Daqing Yin, Xudong Feng

**Affiliations:** ^1^Department of Anesthesiology, The 988th Hospital of Joint Logistic Support Force of Chinese People’s Liberation Army, Zhengzhou, China; ^2^Graduate School of Xinxiang Medical University, Xinxiang, China; ^3^Department of Medical Service, The 988th Hospital of Joint Logistic Support Force of Chinese People’s Liberation Army, Zhengzhou, China

**Keywords:** remote ischemic preconditioning, renal protection, acute kidney injury, repeated tourniquet application, tourniquet

## Abstract

**Objective:**

Limb ischemia–reperfusion injury caused by repeated tourniquet application usually leads to acute kidney injury, adversely affecting patient prognosis. This study aimed to investigate the renoprotective effect of remote ischemic preconditioning (RIPC) in patients undergoing extremity surgery with repeated tourniquet application.

**Methods:**

64 patients were enrolled and randomly divided into an RIPC group and a control group, with 32 patients in each. Pretreatment was administered before surgery, and baseline characteristics were collected. Perioperative surgical characteristics, renal biomarkers, oxidative stress markers, inflammatory factors, and postoperative conditions were recorded.

**Results:**

2 participant were excluded from each group, leaving 30 patients per group. There were no significant differences between the two groups regarding baseline characteristics and perioperative surgical characteristics (*p* > 0.05). Compared to the control group, the RIPC group showed a significant decrease in BUN and SCr at 48 h postoperatively (*p* < 0.05). Levels of Cys-C, [TIMP-2] × [IGFBP-7], KIM-1, IL-18, and NGAL were significantly reduced at the first and second tourniquet releases and at 24 h postoperatively in the RIPC group (*p* < 0.05). From the first tourniquet release to 48 h postoperatively, MDA levels were significantly lower (*p* < 0.05) and SOD levels were significantly higher (*p* < 0.05) in the RIPC group compared to the control group. Postoperative conditions did not differ significantly between the groups.

**Conclusion:**

RIPC effectively mitigated acute kidney injury caused by repeated tourniquet application, offering a robust method for perioperative renal protection in patients undergoing extremity surgery. Future studies should explore the underlying mechanisms and long-term clinical outcomes of RIPC in broader patient populations.

**Clinical trial registration:**

https://www.chictr.org.cn/showproj.html?proj=231266.

## Introduction

Tourniquets are commonly used in orthopedic limb surgery to reduce intraoperative bleeding and improve surgical visibility, allowing for more precise procedures (1). However, tourniquets can also cause complications such as thromboembolism and ischemia–reperfusion injury (IRI) in muscles (2). Ischemia–reperfusion of large muscle tissues can induce rhabdomyolysis by generating inflammatory cells (3). The systemic inflammatory response generated by this process increases vascular permeability, decreases intravascular volume, and activates the renin-angiotensin-aldosterone system. This leads to renal vasoconstriction, reduced renal blood flow and oxygenation, and the generation of free radicals, primarily reactive oxygen species (ROS), which in severe cases can result in acute kidney injury (AKI) (4). The incidence of tourniquet-related AKI ranges from 0.8 to 17.2%, depending on whether patients have risk factors such as diabetes and hypertension (5). Currently, there are no specific measures to prevent or treat tourniquet-induced kidney or other organ damage (6).

Ischemic preconditioning (IPC) involves performing multiple transient cycles of ischemia and reperfusion to the target organ, which makes the organism resistant to further severe injury. This protective phenomenon was first identified in cardiac experiments by Murry et al. (7). A recent systematic review showed that IPC can be applied to the kidney, providing a protective effect (8). Remote ischemic preconditioning (RIPC) involves exposing tissues or organs distant from the target organ (usually upper or lower limbs) to brief cycles of ischemia and reperfusion to minimize subsequent damage to the target organ. Przyklenk et al. conducted the first study on RIPC in 1993 and concluded that RIPC could mitigate myocardial IRI (9). RIPC has the potential to protect a wide range of organs and tissues, including the heart, brain, kidneys, lungs, liver, and skin (10, 11). Multiple meta-analyses have indicated that the incidence of AKI after cardiac surgery is much higher than after myocardial infarction. RIPC before the induction of general anesthesia reduces peak serum creatinine (SCr) and neutrophil gelatinase-associated lipocalin (NGAL) levels and decreases the incidence of AKI by approximately 15% in patients with high-risk factors (12, 13). Studies on the short-term and long-term outcomes of kidney transplantation revealed that preoperative RIPC not only reduced the incidence of acute rejection in recipients but also led to sustained improvements in glomerular filtration rate over a period of five years, prolonging the use of the transplanted kidney (14, 15).

Many studies on RIPC have focused on renal function after cardiac surgery, with few exploring the role of RIPC in renal injury caused by tourniquets, particularly with repeated use (16, 17). Therefore, this study aims to investigate the renoprotective effect of RIPC on repeated tourniquet application in extremity surgery by detecting renal injury biomarkers, oxidative stress markers, and inflammatory factors in the blood. These results may help identify a safe, convenient, and effective perioperative renoprotective measure.

## Materials and methods

### Study design and participants

This prospective, randomized controlled clinical trial investigated the renoprotective effect of RIPC on acute kidney injury caused by repeated tourniquet application in extremity surgery. The study was approved by the Ethics Committee of the 988th Hospital of Joint Logistic Support Force of Chinese People’s Liberation Army (988YY20230041LLSP) and was prospectively registered in the Chinese Clinical Trial Registry (ChiCTR2400088778). Written informed consent was obtained from all participants. Inclusion criteria for the study were patients aged 20–50 years who were scheduled for limb surgery under general anesthesia, with tourniquets applied twice during the operation, each application lasting more than 40 min. Eligible participants had a body mass index (BMI) between 20 and 30 kg/m^2^ and an American Society of Anesthesiologists (ASA) physical status of I-III. Exclusion criteria included individuals with hypertension, diabetes, or other significant comorbidities, those with vascular diseases of the upper extremity, and those with psychiatric disorders. Additionally, patients experiencing serious intraoperative complications such as cardiac arrest or anaphylactic shock, those with bleeding volumes exceeding 800 mL significantly affecting hemodynamic stability, and those who voluntarily withdrew from the study were excluded.

### Randomization and blinding

**Figure 1:**
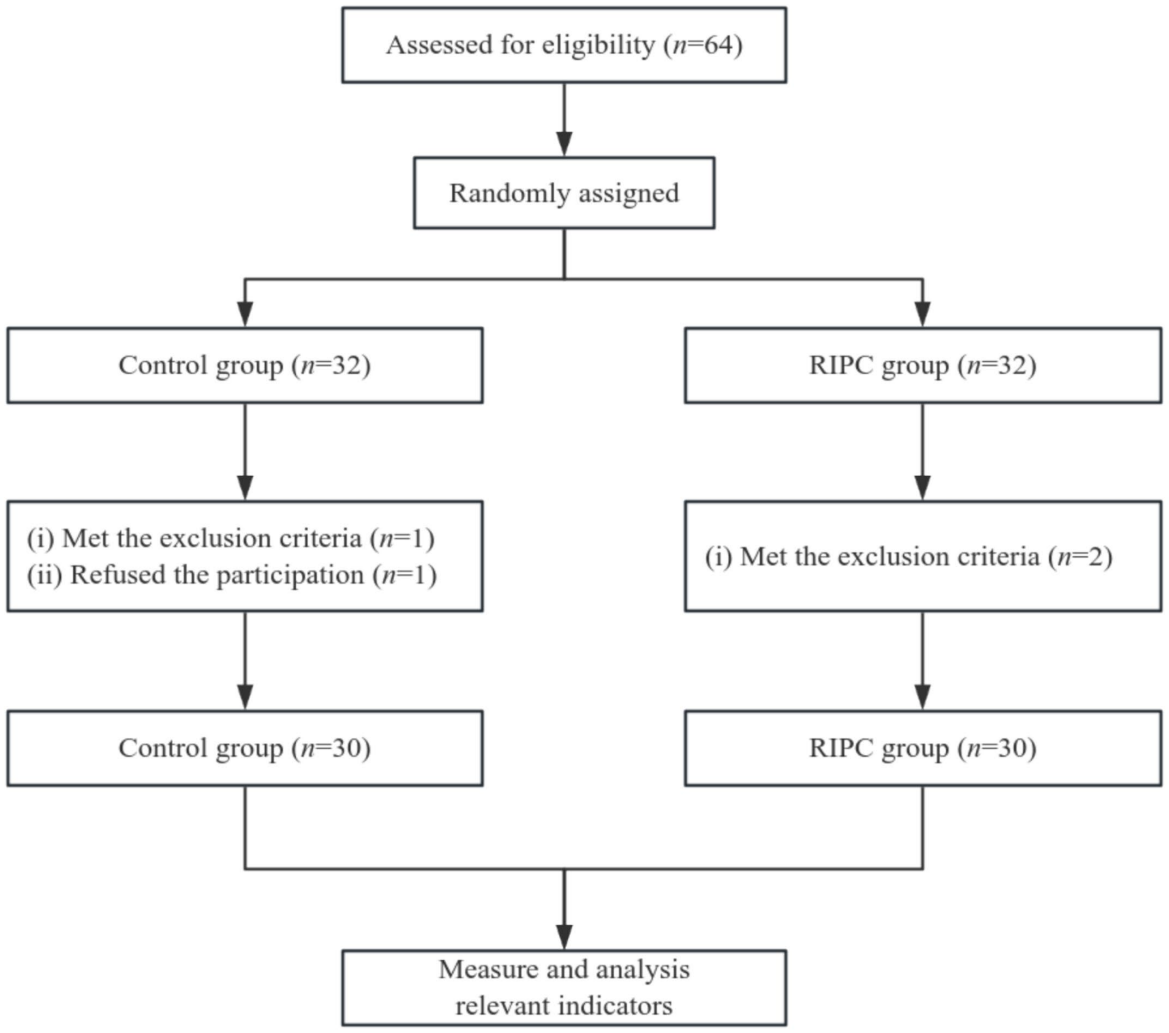
Flow diagram of grouping.

As shown in Figure 1, 64 eligible patients were enrolled and randomly assigned to either the control group (*n* = 32) or the RIPC group (*n* = 32). One patient in the control group met the exclusion criteria, and another refused participation; two patients in the RIPC group met the exclusion criteria. Thus, 30 subjects remained in each group. Eligible patients were randomized in a 1:1 ratio one hour before anesthesia and numbered sequentially. A well-trained team member performed the preconditioning. The operators, subjects, anesthesiologists, surgeons, and data processors were all blinded to the group assignments.

### Procedural protocol

Upon admission to the operating room, all eligible patients underwent artery puncture and cannulation under local anesthesia with lidocaine, followed by continuous vital signs monitoring. All patients received a standardized anesthesia induction regimen: penehyclidine hydrochloride 0.4 mg, flurbiprofen axetil 50 mg, etomidate 0.3 mg/kg, sufentanil citrate 0.4 μg/kg, and rocuronium bromide 0.5 mg/kg. After correct placement of the laryngeal mask, mechanical ventilation was initiated. Anesthesia was maintained with a continuous infusion of propofol (35–45 μg/kg/min) and remifentanil (0.35–0.45 μg/kg/min).

In the RIPC group, an inflatable pressure tourniquet was placed 2.5 cm above the elbow of the patient’s healthy upper limb one hour before surgery. The tourniquet was inflated to 200 mmHg for three cycles of 5 min inflation and 5 min deflation. In the control group, a tourniquet was placed similarly but without preconditioning. Vital signs were monitored to ensure stability during the operation, and the laryngeal mask was removed after the patient fully recovered.

### Outcome measures

Primary outcomes included renal tubular injury markers (tissue inhibitor of metalloproteinases-2 [TIMP-2], insulin-like growth factor-binding protein-7 [IGFBP-7], kidney injury molecule-1 [KIM-1]), glomerular filtration function (blood urea nitrogen [BUN], SCr, serum cystatin-C [Cys-C]), and markers of oxidative stress and inflammation (malondialdehyde [MDA], superoxide dismutase [SOD], interleukin-18 [IL-18], NGAL), and the calculation of [TIMP-2] × [IGFBP-7]. These indicators were all collected at five time points: before anesthesia induction (T0), at the first tourniquet release (T1), at the second tourniquet release (T2), 24 h postoperatively (T3), and 48 h postoperatively (T4).

Secondary outcomes included baseline characteristics (age, sex, BMI, ASA status, preoperative SCr), perioperative surgical characteristics (surgery duration, first and second tourniquet durations, infusion volume, urine volume, and bleeding volume), and postoperative outcomes (intensive care unit [ICU] occupancy, nephrology consultations, and length of hospital stay).

### Statistical analysis

Based on pre-trial results, we calculated that 50 patients were needed to achieve adequate power, with a 20% anticipated loss to follow-up, resulting in 64 patients recruited. Data were analyzed using GraphPad Prism 9.5. Normality was assessed using the Shapiro–Wilk test. Normally distributed data were expressed as mean ± standard deviation ( $\bar{x\;}$ ±s). For normally distributed data with equal variances, a *t*-test was used to compare between the two groups. For comparisons of measurements at different time points, repeated measures analysis of two-way ANOVA was employed. Categorical data were presented as percentages and compared using the chi-square (χ^2^) test. A significant level of *α* = 0.05 was used, with *p* < 0.05 considered statistically significant.

## Results

### Baseline characteristics

**Figure 2:**
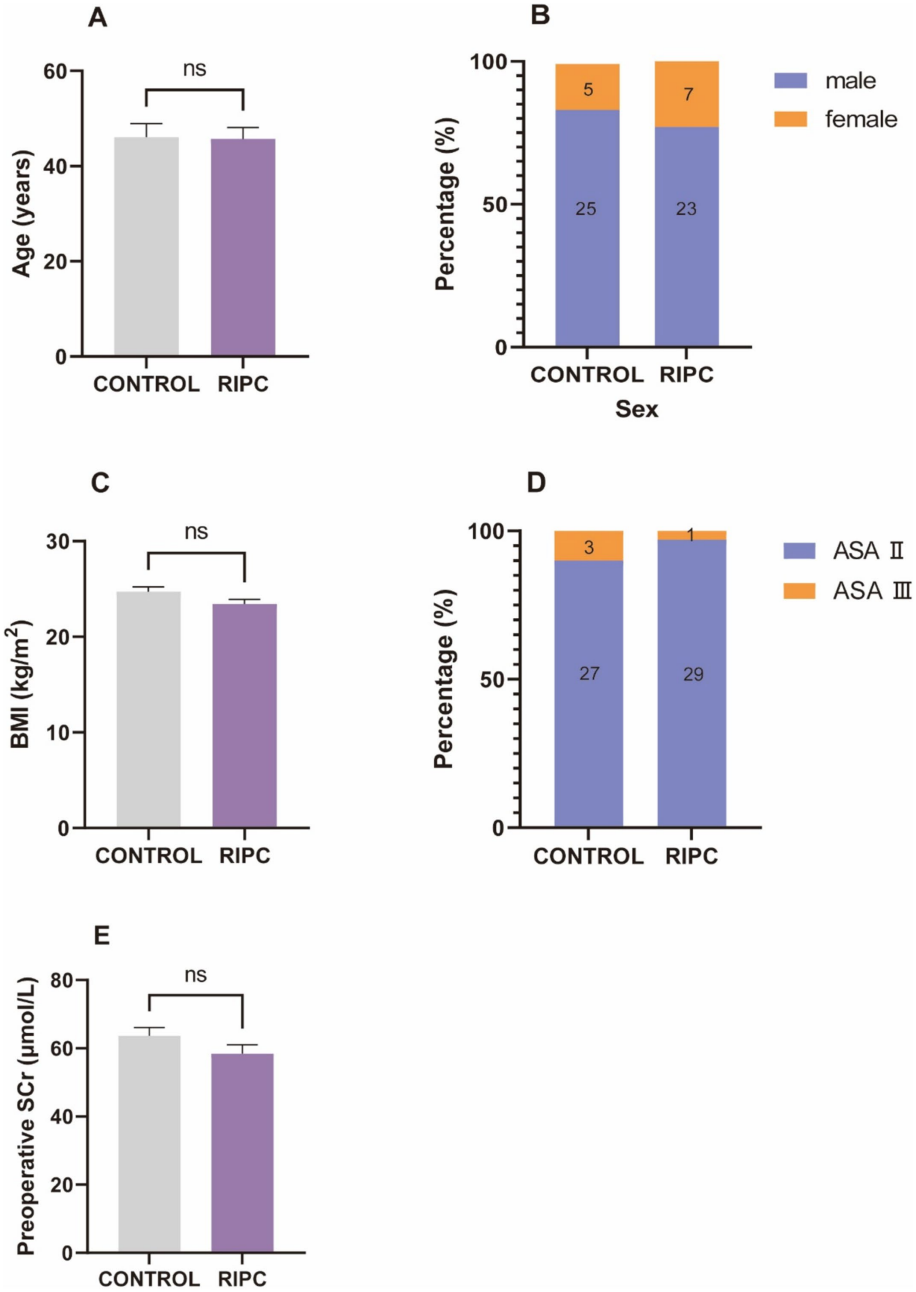
Baseline characteristics. **(A)** The mean age of control group was 46.07 ± 15.68, the mean age of RIPC group was 45.73 ± 12.98, there was no significant difference in age between two groups. **(B)** There were 25 male and 5 female in control group, 23 male and 7 female in RIPC group, no significant difference in gender between two groups. **(C)** The mean BMI of control group was 24.72 ± 2.66, the mean BMI of RIPC group was 23.45 ± 2.59, there was no significant difference in BMI between two groups. **(D)** There were 27 ASA II and 3 ASA III in control group, 29 ASA II and 1 ASA III in RIPC group, no significant difference in ASA classification between two groups. **(E)** The mean age of control group was 63.63 ± 13.59, the mean age of RIPC group was 58.43 ± 14.06, there was no significant difference in preoperative SCr levels between two groups.

A total of 60 patients were included in this study, comprising 48 males and 12 females. There were no statistically significant differences between the RIPC group and the control group in terms of gender, age, BMI, ASA classification, and preoperative SCr levels (all *p* > 0.05, Figure 2).

### Perioperative surgical characteristics

As presented in Table 1, there were no significant differences between the RIPC group and the control group regarding surgery duration, the first tourniquet duration, the second tourniquet duration, infusion volume, urine volume, and bleeding volume (all *p* > 0.05).

**Table 1:** Perioperative surgical characteristics.

Characteristics	Control group (*n* = 30)	RIPC group (*n* = 30)	t	*p* value
Surgery duration (min)	226.17 ± 105.45	217.67 ± 114.43	0.30	0.77
First tourniquet duration (min)	65.87 ± 19.26	56.60 ± 17.90	1.93	0.06
Second tourniquet duration (min)	61.47 ± 18.22	62.93 ± 16.90	0.32	0.75
Infusion volume (mL)	1813.33 ± 621.72	1823.67 ± 914.19	0.05	0.96
Urine volume (mL)	573.33 ± 663.16	527.67 ± 664.00	0.27	0.79
Bleeding volume (mL)	147.97 ± 198.31	158.70 ± 191.18	0.02	0.83

### Comparison of perioperative renal function

The renoprotective effect of RIPC was assessed by comparing glomerular and tubular function in patients undergoing extremity surgery with repeated tourniquet application. As shown in Table 2, the RIPC group exhibited significantly lower BUN and SCr levels at T4 compared to the control group (*p* < 0.05). Additionally, the RIPC group had significantly lower Cys-C levels at T1, T2, and T3 (*p* < 0.05), suggesting reduced glomerular damage. Furthermore, the levels of TIMP-2, IGFBP-7, [TIMP-2] × [IGFBP-7], and KIM-1 at T1, T2, and T3 were significantly lower in the RIPC group compared to the control group (all *p* < 0.05), indicating that RIPC attenuated tubular damage caused by repeated tourniquet application.

**Table 2:** Comparison of perioperative renal function ( $\bar{x}$ ±s).

Groups	Kidney function	Time points				
		T0	T1	T2	T3	T4
Control group (*n* = 30)	BUN (mg/dL)	19.96 ± 4.54	28.24 ± 2.95	36.79 ± 5.33	31.19 ± 3.78	21.61 ± 4.05
	SCr (μmol/L)	86.54 ± 11.63	104.87 ± 9.44	130.92 ± 10.40	136.83 ± 11.02	90.59 ± 10.74
	Cys-C (mg/L)	0.63 ± 0.24	1.36 ± 0.25	2.00 ± 0.39	2.23 ± 0.52	1.15 ± 0.43
	TIMP-2 (μg/L)	0.63 ± 0.12	1.16 ± 0.28	1.53 ± 0.28	1.64 ± 0.32	0.88 ± 0.22
	IGFBP-7 (μg/L)	25.08 ± 7.60	53.02 ± 8.82	68.95 ± 8.11	64.88 ± 13.34	36.26 ± 7.10
	[TIMP-2] × [IGFBP-7] (μg/L)^2^	15.65 ± 4.90	62.26 ± 20.43	106.07 ± 25.72	106.50 ± 29.57	32.27 ± 12.01
	KIM-1 (ng/L)	11.58 ± 3.63	23.84 ± 6.25	32.99 ± 5.62	38.09 ± 6.44	17.67 ± 4.90
RIPC group (*n* = 30)	BUN (mg/dL)	20.11 ± 3.41	26.55 ± 4.05	36.79 ± 8.01	29.95 ± 3.71	16.92 ± 3.57^c^
	SCr (μmol/L)	82.71 ± 15.12	103.61 ± 12.92	125.26 ± 12.31	133.16 ± 12.09	73.65 ± 15.62^c^
	Cys-C (mg/L)	0.64 ± 0.25	1.11 ± 0.32^b^	1.66 ± 0.43^a^	1.86 ± 0.47^a^	1.01 ± 0.25
	TIMP-2 (μg/L)	0.64 ± 0.16	0.97 ± 0.22^a^	1.25 ± 0.24^c^	1.31 ± 0.30^c^	0.77 ± 0.24
	IGFBP-7 (μg/L)	24.07 ± 8.50	43.28 ± 12.12^b^	58.56 ± 7.38^c^	56.32 ± 11.35^a^	33.19 ± 7.15
	[TIMP-2] × [IGFBP-7] (μg/L)^2^	15.25 ± 5.94	41.55 ± 13.56^c^	73.94 ± 19.32^c^	75.66 ± 27.55^c^	25.54 ± 9.12
	KIM-1 (ng/L)	11.53 ± 3.38	20.05 ± 4.41^a^	29.19 ± 3.17^a^	32.11 ± 6.40^b^	15.21 ± 4.06

a *P < 0.05 compared to the control group.*

b *P < 0.01 compared to the control group.*

c *P < 0.001 compared to the control group.*

### Changes in oxidative stress and inflammatory factors

We further investigated whether RIPC mitigated oxidative stress and inflammatory responses induced by repeated tourniquet application. As shown in Table 3, the RIPC group exhibited lower MDA levels and higher SOD levels at T1, T2, T3, and T4 compared to the control group (all *p* < 0.05). At T1 and T2, the SOD level decreased in both groups, but the reduction was less pronounced in the RIPC group (*p* < 0.05). At T3 and T4, the SOD level increased in both groups, with a greater elevation observed in the RIPC group (*p* < 0.05). Additionally, the RIPC group had significantly lower levels of IL-18 and NGAL at T1, T2, and T3 (all *p* < 0.05). These findings indicated that RIPC effectively attenuates oxidative stress and inflammatory responses associated with repeated tourniquet application.

**Table 3:** Changes in oxidative stress and inflammatory factors ( $\bar{x}$ ±s).

Groups	Characteristics	Time points				
		T0	T1	T2	T3	T4
Control group (*n* = 30)	MDA (nmol/mL)	14.62 ± 7.79	48.78 ± 12.66	73.29 ± 20.98	82.39 ± 22.71	38.62 ± 13.89
	SOD (U/mL)	241.68 ± 51.12	140.00 ± 33.67	113.67 ± 25.86	139.55 ± 27.45	172.77 ± 36.71
	IL-18 (ng/L)	132.45 ± 30.80	302.04 ± 46.86	393.48 ± 46.69	430.37 ± 49.93	228.97 ± 39.90
	NGAL (μg/L)	24.15 ± 5.38	34.92 ± 5.13	45.00 ± 5.83	48.45 ± 5.43	32.75 ± 10.57
RIPC group (*n* = 30)	MDA (nmol/mL)	13.89 ± 6.22	38.40 ± 12.45^a^	56.48 ± 16.69^b^	65.16 ± 18.15^b^	28.62 ± 13.16^a^
	SOD (U/mL)	245.87 ± 58.71	171.74 ± 42.58^a^	134.58 ± 31.73^a^	161.64 ± 32.89^a^	201.30 ± 40.11^a^
	IL-18 (ng/L)	130.78 ± 27.30	237.74 ± 57.68^c^	313.46 ± 59.22^c^	351.65 ± 49.34^c^	205.21 ± 36.10
	NGAL (μg/L)	22.12 ± 6.18	30.98 ± 5.80^a^	37.22 ± 5.27^c^	40.41 ± 5.47^c^	29.04 ± 6.73

a *P < 0.05 compared to the control group.*

b *P < 0.01 compared to the control group.*

c *P < 0.001 compared to the control group.*

### Postoperative outcomes

Finally, we compared the postoperative outcomes between the two groups. As shown in Table 4, there were no significant differences between the RIPC group and the control group in terms of ICU occupancy, nephrology consultations, and postoperative hospital stay (all *p* > 0.05).

**Table 4:** Postoperative situation.

Characteristics	Control group (*n* = 30)	RIPC group (*n* = 30)	t/z	*P* value
ICU occupancy (%)	4 (13.33)	1 (3.33)	1.96	0.16
Nephrology consultation (%)	1 (3.33)	0 (0.00)	1.02	0.31
Postoperative hospital stay (days)	20.43 ± 22.09	12.07 ± 8.81	1.93	0.06

## Discussion

Tourniquets are commonly used in extremity surgeries for orthopedic patients to assist surgeons in better completing the surgery. However, muscle IRI from prolonged tourniquet use can lead to tissue and organ damage and, in severe cases, multiple systemic organ failure (18). RIPC has emerged in recent years as a convenient, non-invasive, and inexpensive protective method applicable to a wide range of organs. Current studies have found that preoperative use of RIPC reduces the incidence of postoperative AKI without adverse events (19). Our study found that RIPC could attenuate AKI induced by repeated tourniquet application in extremities, which may through inhibiting ROS production, oxidative stress and inflammatory responses, and inducing transient cell cycle arrest. This might provide a new strategy for mitigating perioperative AKI in the clinic.

The exact mechanism by which repeated tourniquet application and limb IRI lead to AKI is not fully elucidated. Most studies suggest that this phenomenon may be caused by chronic hypoperfusion and hypoxia of the kidney, related to the production of ROS such as superoxide, hydrogen peroxide, and hydroxyl radicals, as well as the involvement of inflammatory cells (20). During the ischemic phase, decreased aerobic metabolism and increased anaerobic metabolism led to the depletion of adenosine triphosphate (ATP), disruption of intracellular redox homeostasis, accumulation of acidic metabolites, and the failure of ATP-dependent sodium-potassium pumps and sodium-calcium exchangers. These disruptions result in increased intracellular Na^+^ and Ca^2+^, ultimately causing apoptosis (21). Superoxide is a key mediator of cell necrosis and tissue injury during reperfusion (22). It is produced when xanthine is oxidized to uric acid. During ischemia, decreased ATP leads to increased hypoxanthine, which is then oxidized during reperfusion to produce large amounts of superoxide (23, 24). SOD is an antioxidant enzyme that converts superoxide into oxygen and hydrogen peroxide, reducing ROS levels and protecting organs from oxidative damage. It is one of the most crucial antioxidant enzymes in cells (25). Researches have shown that SOD can protect the kidneys from chronic ischemic injury and prevent renal insufficiency through its antioxidant, vasodilatory, and antihypertensive effects (26). ROS-induced cell damage initiates lipid peroxidation, with MDA being a harmful end product that can result in cell damage and apoptosis, often used to indicate ROS levels (27, 28). Large amounts of ROS released into the blood during reperfusion elicit an inflammatory response, with the activation of inflammatory pathways and recruitment of inflammatory cells being early responses to renal injury.

To date, no single definitive measure has been found to prevent or mitigate AKI. Therefore, it is significant to explore simple methods to attenuate AKI caused by repeated tourniquet application and limb IRI by suppressing ROS levels and inflammatory responses. RIPC involves exposing the body to brief cycles of ischemia and reperfusion by pretreating organs and tissues far from the target organ, which has been shown to attenuate tissue and organ damage (29). The exact mechanism by which RIPC protects the kidney is not fully understood. However, most studies suggest it may involve the production of nitric oxide or nitrite, the release of damage-associated molecular patterns, activation of transient cell cycle arrest in renal tubular epithelial cells, and the clearance of damaged mitochondria via mitochondrial autophagy (30). RIPC may protect the kidney by activating natural defenses that cause renal tubular epithelial cells to undergo transient cell cycle arrest, enabling them to withstand subsequent oxidative stress or IRI. Many studies have identified TIMP-2 and IGFBP-7 as markers of cell cycle arrest (31). Alexander et al. revealed that RIPC could reduce [TIMP-2] × [IGFBP-7] levels in patients at high risk for AKI during cardiac surgery, contributing to a significant reduction in the incidence of AKI (12). Our study also confirmed that [TIMP-2] × [IGFBP-7] and KIM-1 levels were significantly reduced at T1, T2, and T3 in the RIPC group, suggesting that RIPC may attenuate tubular injury caused by repeated tourniquet application by inducing transient tubular epithelial cell cycle arrest.

Cys-C is a biomarker that is freely filtered through the glomeruli and completely absorbed by renal tubular epithelial cells, with minimal influence from external disturbances. Therefore, it has higher sensitivity and specificity in predicting AKI compared to SCr and BUN (32). Kasepalu et al. demonstrated that SCr, urea, and Cys-C were significantly reduced in the RIPC group of patients undergoing lower extremity revascularization (33). Similarly, our study found that the RIPC group exhibited lower levels of SCr and BUN at T4, and decreased level of Cys-C at T1, T2, and T3, indicating that RIPC could mitigate the effects of limb IRI induced by repeated tourniquet application on postoperative glomerular filtration function.

Oxidative stress markers and inflammation levels were also examined in this study. NGAL, primarily secreted by immune cells such as neutrophils, macrophages, and dendritic cells, is produced in response to inflammation and released after tubular injury during renal regeneration. It can be used as a predictor of renal function progression concerning renal failure (34). IL-18, an inflammatory factor released after acute ischemic injury, is considered an early diagnostic marker for AKI. Guo et al. demonstrated that RIPC attenuated postoperative NGAL levels compared to the control group (35). Luan et al. showed that IL-18 knockout ameliorated tubular injury and limited the progression of AKI to chronic kidney disease (36). Similarly, our study found that IL-18 and NGAL levels at T1, T2, and T3 were significantly lower in the RIPC group. Furthermore, RIPC also demonstrated the ability to mitigate oxidative stress in AKI induced by limb IRI by reducing MDA levels and increasing SOD levels in our study. These findings suggest that the protective effect of RIPC may be mediated through the inhibition of oxidative stress and inflammatory responses in AKI induced by limb IRI.

This study has several limitations. Firstly, it was conducted as a single-center study with a limited sample size, which may affect the generalizability of the results. Future studies with larger, multicenter cohorts are necessary to validate these findings. Secondly, the antioxidant and renoprotective properties of anesthetic drugs were not considered in this study. Although we designed the study to minimize the potential impact of anesthetics on the outcomes, future research should explore the synergistic effects of anesthetic agents and RIPC on renal protection. Despite these limitations, our study demonstrates that RIPC is a promising and non-invasive method to attenuate AKI induced by repeated tourniquet use in extremity surgeries. The findings suggest potential clinical applications for improving perioperative renal outcomes, highlighting the need for further investigation into RIPC’s protective mechanisms and broader applicability. Future research should focus on elucidating the precise mechanisms underlying RIPC’s renoprotective effects and evaluating its long-term clinical benefits across diverse surgical populations.

## Data Availability

The raw data supporting the conclusions of this article will be made available by the authors, without undue reservation.
